# Integrated biosynthesis of the lignan (-)-pluviatolide in resting and growing *E. coli* cells

**DOI:** 10.3389/fbioe.2026.1716646

**Published:** 2026-01-16

**Authors:** Ronja Knöfel, Jonas Barsig, Philipp A. Bechtold, Cigdem Günes, U. Joost Luelf, Vlada B. Urlacher

**Affiliations:** Institute of Biochemistry, Heinrich Heine University Düsseldorf, Düsseldorf, Germany

**Keywords:** growing cells, multi-enzyme cascade, plant lignans, recombinant *E. coli*, resting cells

## Abstract

Lignans exhibit a wide range of useful bioactivities. A key intermediate in their biosynthesis in plants is (−)-pluviatolide, which directs the pathway towards various high-value lignans like (−)-podophyllotoxin - the precursor of the clinically relevant antitumor drugs etoposide and teniposide. In an attempt to develop more sustainable ways for the production of lignans, which are traditionally isolated from plants, we previously established a heterologous biosynthesis of (−)-pluviatolide in *Escherichia coli*, in which recombinant genes were expressed from multiple plasmids. In this study, the genes encoding the four-enzyme, four-step reaction cascade from (+)-pinoresinol to (−)-pluviatolide were integrated into the chromosome of *E. coli* C41(DE3). The plasmid-based and plasmid-free *E. coli* strains were compared in resting and growing cell approaches. The performance of the plasmid-free recombinant system was similar to that of the plasmid-based system, regardless of the approach tested. The addition of glycerol and glucose as energy and carbon sources enhanced the productivity towards (−)-pluviatolide. LC-MS analysis revealed complete conversion of the substrate (+)-pinoresinol and the formation of (−)-pluviatolide with 99% product ratio in resting cells and 92% in growing cells.

## 1 Introduction

**FIGURE 1:**
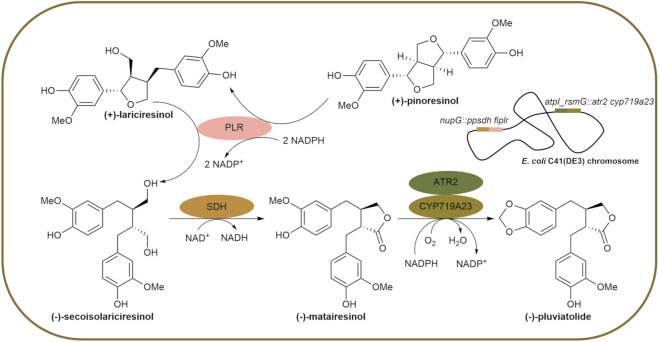
Recombinant *Escherichia coli* C41(DE3) cell for the reconstituted plant lignan pathway from (+)-pinoresinol to (−)-pluviatolide with genes integrated into the locus *nupG* and the intergenic region *atpI_rsmG*. PLR: pinoresinol-lariciresinol reductase from *Forsythia intermedia*, SDH: secoisolariciresinol dehydrogenase from *Podophyllum pleianthum*, CYP719A23: cytochrome P450 monooxygenase from *Podophyllum hexandrum*, ATR2: cytochrome P450 reductase from *Arabidopsis thaliana*.

Lignans are a group of natural compounds sharing the structural motif of *β*-*β′*-linked phenylpropanoid dimers and are associated with a large variety of bioactivities and health benefits, like antitumor, antiviral, anti-inflammatory, antioxidant or neuroprotective effects (Zálešák et al., 2019; Wang et al., 2022). For example, the dibenzylbutyrolactone lignan (−)-pluviatolide has been described to display anti-spasmodic and cytotoxic activity (Zhang et al., 2008). More importantly, it is a crucial cross-road lignan, serving as an entry point into distinct biosynthetic pathways towards high-value lignans such as (−)-podophyllotoxin. The latter is the direct precursor of the clinically relevant chemotherapeutics etoposide and teniposide. These drugs are primarily obtained by chemical modification of (−)-podophyllotoxin extracted from the endangered *Podophyllum* species like *Podophyllum hexandrum* (Shah et al., 2021). However, long generation cycles, low lignan content, laborious extraction and purification and finally, the requirement for uprooting the plants render this procedure inefficient, necessitating different strategies (Singh et al., 2024). One promising alternative is the heterologous microbial production, as the natural lignan pathway to (−)-podophyllotoxin has been mostly deciphered, with only the final step remaining elusive (Dinkova-Kostova et al., 1996; Xia et al., 2001; Marques et al., 2013; Lau and Sattely, 2015). To this end, previous studies in our group have established heterologous pathways to (−)-deoxypodophyllotoxin from (−)-matairesinol, and to (−)-pluviatolide from (+)-pinoresinol in resting recombinant *E. coli* cells (Decembrino et al., 2020; Decembrino et al., 2021). The latter biosynthetic pathway was reconstituted in a two-plasmid-based approach. Initially, (+)-pinoresinol is reduced sequentially to (+)-lariciresinol and (−)-secoisolariciresinol with both steps catalyzed by pinoresinol-lariciresinol reductase (PLR). Then, secoisolariciresinol dehydrogenase (SDH) catalyzes the intramolecular formation of a lactone ring leading to (−)-matairesinol, which is further converted by the cytochrome P450 monooxygenase CYP719A23, generating the methylenedioxy bridge in (−)-pluviatolide (Figure 1). The latter step has been identified as limiting the cascade (Decembrino et al., 2020). CYP719A23, which catalyzes this step, is a heme b-containing enzyme that relies on redox partner proteins for its activity. Since the physiological redox partner for CYP719A23 has not yet been identified, several heterologous redox partner proteins have been tested *in vitro* and in resting *Escherichia coli* cells harboring two plasmids (Decembrino et al., 2020). The co-expression of the *cyp* gene with the gene of the cytochrome P450 reductase ATR2 of *Arabidopsis thaliana* resulted in the highest product titers when normalized to cell weight. These observations allowed us to conclude that, in the described system, the redox partner choice is important, but the expression level of CYP719A23 is essential for high cell performance. In this context, codon optimization and a thorough engineering of the membrane-bound N-terminus of CYP719A23 were also carried out (Decembrino et al., 2020).

Various strategies can be used to achieve balanced expression of all enzymes and thus optimize metabolic flow through a synthetic metabolic pathway (Lin and Tao, 2017). In this context, the growth phase for biomass production and enzyme expression can be separated from substrate conversion using resting cells. Resting cells are used under non-growing conditions, usually resuspended in buffer. Thus, they have a lower metabolic energy demand, and carbon and energy sources that are otherwise used for growth can be channeled into the biotransformation. Furthermore, they offer simplified downstream purification due to fewer metabolic intermediates and by-products, and can be reused (de Carvalho, 2017; Nain et al., 2024). However, resting cells do have a reduced capacity to recycle cofactors, which may become critical for cofactor-dependent reactions (Hibi et al., 2007; Zehentgruber et al., 2010). On the other hand, cell growth and substrate conversion can be carried out in parallel in a fermentation-like fashion within growing cells. They are metabolically active and proliferate and thus can regenerate cofactors and enzymes by continuous expression (Hou et al., 2016). Growing cells might be more robust to biocatalysis, oxidative stress and product inhibition (Bertelmann and Bühler, 2024). Furthermore, growing cells do not need to be harvested before biotransformation, which makes the entire process less time-consuming and resource-intensive. However, there is competition for carbon and energy sources, cofactors and the cellular machinery between biomass production and protein overexpression and substrate conversion (Julsing et al., 2012). Clearly, both approaches - resting and growing cells - should be compared to find the most optimal system for each specific biotransformation or reaction cascade.

While plasmids are commonly used for recombinant protein expression in *E. coli* due to their easy genetic manipulation and scalable copy numbers (Englaender et al., 2017; Sjöberg et al., 2019), episomal expression has several drawbacks, like possible cell-to-cell variability, reduced cell growth, and higher costs due to the need for antibiotics or other selection markers (Saleski et al., 2021). In addition, the requirement for compatible vectors limits the number of enzymes that can be co-expressed within one cell, and thus impedes the reconstitution of long and complex recombinant pathways for the production of value-added compounds (Sjöberg et al., 2019). Chromosomal integration of recombinant genes, on the other hand, has been reported to reduce the metabolic burden and to lead to inherent expression stability in the host cells, favorable for long-term fed-batch fermentations (Striedner et al., 2010; Englaender et al., 2017; Ou et al., 2018; Wang et al., 2024). Consequently, significant efforts are being made to implement biosynthetic pathways into the chromosomes of producing microorganisms. Examples for *E. coli* include the biosynthesis of anthranilate (Kim et al., 2023), salvianic acid A (Zhou et al., 2017), *β*-carotene (Ye et al., 2016) or lycopene (Chen et al., 2013).

In this study, the genes encoding four enzymes of the four-step reaction cascade from (+)-pinoresinol to (−)-pluviatolide (Figure 1) were integrated into the chromosome of *E. coli* C41(DE3). Two *E. coli* strains were compared regarding their capacity to produce the final product (−)-pluviatolide: one with two plasmids (hereinafter referred to as episomal system) and one with four genes integrated into the chromosome (hereinafter referred to as chromosomal system). The strains were studied in resting and growing cell approaches. As determined via LC-MS based on a calibration curve, the final product ratio of (−)-pluviatolide was 99% (corresponding to 71 mg/L) and 92% (66 mg/L) in resting and growing cells, respectively.

## 2 Materials and methods

### 2.1 Cloning and chromosomal integration

The genes *ppsdh* and *fiplr* were transferred via restriction-ligation from the pCDFDuet vector (Decembrino et al., 2020) to pACYCDuet (Novagen) in an iterative manner using NcoI and HindIII (for *ppsdh*) and NdeI and XhoI (for *fiplr*) and T4 ligase. Chromosomal integration of the genes was conducted as previously described, adapting the toolbox designed for the straightforward integration of the multiple cloning sites of pETDuet vectors (Luelf et al., 2023; Luelf et al., 2024). In brief, homology arms to the target loci *nupG* and *atpI_rsmG* were amplified with Taq polymerase from *E. coli* BL21(DE3), which shows 100% sequence homology to *E. coli* C41(DE3) in the target areas. The two multiple cloning sites (MCS) of pETDuet (Novagen) were amplified with corresponding overlaps using Phusion polymerase. Then, the PCR products were assembled using Fusion PCR. pgRNA plasmids with the N_20_ targeting sequences for the loci *nupG* and *atpI_rsmG* were linearized by PCR and the MCS fragment was inserted into the linearized backbone via Gibson assembly (Gibson et al., 2009), creating the plasmids pgRNADuet_nupG and pgRNADuet_atpI_rsmG. The genes *fiplr* and *ppsdh* or *cyp719a23* and *atr2* were integrated into the respective pgRNADuet vectors by restriction ligation cloning using NdeI and XhoI or NotI and NcoI, respectively. For chromosomal integration, electrocompetent cells of *E. coli* C41(DE3) carrying the plasmid pEcCas for expression of *cas9* and λ-Red genes (Li et al., 2021) were prepared and transformed with 100 ng of the pgRNADuet plasmids by electroporation (MicroPulser, Bio-Rad, 2 mm gap electroporation cuvette). Correct insertion of the genes was verified by colony PCR with primers binding outside of the respective homology arms of the target locus in the *E. coli* chromosome, and subsequent Sanger sequencing (Eurofins Genomics). That way, it is excluded that the presence of the donor plasmid would lead to false-positive results. Finally, plasmid curing was performed sequentially in 5 mL overnight cultures in LB medium by the addition of l-rhamnose (10 mM) to lose pgRNADuet or sucrose (5% (w/v)) to remove pEcCas, respectively.

### 2.2 Cultivation and determination of CYP450 concentration

The expression was carried out in 100 mL TB (terrific broth) medium, supplemented for plasmid-containing strains with the required antibiotics (100 μg/mL ampicillin, 34 μg/mL chloramphenicol). Main cultures were inoculated with 1 mL of an overnight culture (prepared in LB (lysogeny broth) medium) and incubated at 37 °C, 180 rpm until an OD_600_ of 0.6 was reached. Expression was induced with 0.5 mM isopropyl-β-D-1-thiogalactopyranoside (IPTG), and 0.5 mM 5-aminolevulonic acid (5-ALA) and 0.1 mM FeSO_4_ were added to support heme production. Then, the cultures were incubated at 25 °C, 120 rpm for 48 h. Cells were harvested by centrifugation (3,900 xg, 15 min, 4 °C) and washed once with potassium phosphate buffer (KPi, 50 mM, pH 7.5). The pellet was resuspended in 5 mL KPi buffer. To determine the concentration of the functional heme-containing CYP450, CO-difference spectra were recorded in cell lysates and CYP concentrations were calculated using the extinction coefficient ε_490-450_ = 91 mM^-1^ cm^-1^ as described elsewhere (Omura and Sato, 1964). CYP450 concentrations were determined in biological triplicate.

### 2.3 Biotransformation with resting cells

The expression was carried out in 50 mL TB medium as described above. Cells were harvested after 48 h by centrifugation (3,900 xg, 30 min, 4 °C), washed with KPi buffer and the pellets were frozen at −20 °C until use. Before the biotransformation, cells were normalized to 70 g/L cell wet weight (cww), if not stated otherwise, in resuspension buffer (50 mM KPi, pH 7.5 or KPi supplemented with 400 mM glycerol or 200 mM glucose). 10 mL of the cell suspensions were transferred to 100 mL baffled shake flasks and 0.1 mM IPTG was added. Biotransformation was started by adding (+)-pinoresinol (PhytoLab) from a stock solution in DMSO to a final concentration of 200 μM and 2% DMSO. The cells were incubated in an orbital shaker (Multitron, Infors HT) at 25 °C, 250 rpm. Samples (500 µL each) were taken after 10, 30, 45, 60 and 120 min for product quantification. Biotransformations were carried out in biological triplicate.

### 2.4 Whole-cell catalysis in growing cells

Cells were grown in 50 mL TB medium until OD_600_ = 0.6 and then expression was induced as described above. After incubation at 25 °C, 120 rpm for 2 h, 10 mL of the cultures were transferred to 100 mL baffled shake flasks. The biotransformation was initiated with the addition of 200 µM (+)-pinoresinol and 2% DMSO and performed at 25 °C, 250 rpm for 46 h. Samples (500 µL each) were taken after 4, 22 and 46 h for product quantification, if not stated otherwise. Biotransformations were carried out in biological duplicate or triplicate.

### 2.5 Product analysis

For analysis, 500 µL samples were drawn from the respective cell cultures and a spatula tip of NaCl (∼10 mg) as well as (+)-sesamin as internal standard (final concentration 200 µM) were added. The samples were extracted twice with 500 µL ethyl acetate each: To do so, the suspension was vortexed for 2 min, centrifuged for 4 min (12,300 xg, RT) and 300 µL organic phase was transferred to a fresh tube. The pooled organic phases were removed under reduced pressure (RVC 2–25 CDplus, Christ) and the pellet was resuspended in 50 µL methanol. Samples were analyzed by liquid chromatography coupled with mass spectrometry (LC-MS, LCMS-2020 system, Shimadzu). The separation of analytes occurred on a reversed-phase C18 column (Chromolith Performance RP-18e, 100 mm × 4.6 mm, Merck) using a mobile phase gradient of H_2_O and methanol with 0.1% formic acid (Supplementary Table S5). The column temperature was 30 °C, the flow rate was 0.8 mL/min and 1 µL of sample was injected. Products were monitored both via photo diode array detector at 280 nm and via mass spectrometry after electrospray ionization (ESI) and atmospheric pressure chemical ionization (APCI). Analysis was conducted in positive ion mode in a mass-to-charge (m/z) range of 159–1,000 m/z with a scan speed of 3,750 u/sec. Product distribution was determined from the total ion chromatogram (TIC) as follows, with P_area_ and S_area_ referring to the peak areas of products and substrate, respectively:

$$Product\;distribution\;\left[\%\right]={\;P}_{area}\;/\;\Sigma \;\left(S_{area}+P_{area}\right)\;\cdot \;100.$$

Products were identified based on retention times and characteristic m/z fragments compared to authentic references (Supplementary Figure S1) and our previous study (Decembrino et al., 2020). The color palette used for the data visualization was adapted from the batlow color scheme (Crameri, 2023). For the quantitative analysis of (−)-pluviatolide and (−)-matairesinol, internal standard calibration curves were generated from triplicates (Supplementary Figure S2). Therefore, these analytes were added to 500 μL TB medium with final concentrations in a range of 10–200 μM, and extracted afterwards as described above.

## 3 Results

### 3.1 Gene integration into the *Escherichia coli* chromosome

**FIGURE 2:**
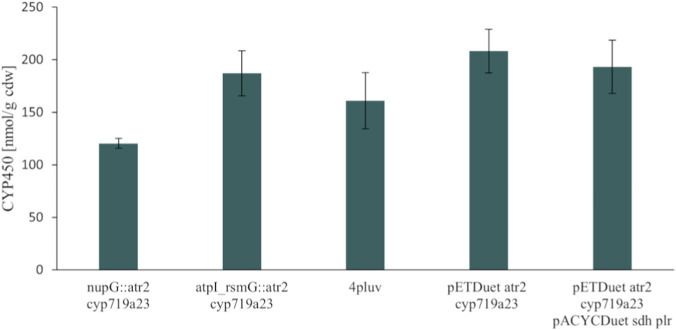
CYP719A23 concentrations determined from the CO-difference spectra of cell lysates after 48 h of expression. The genes encoding for the CYP450 enzyme and the reductase ATR2 were integrated into the loci *nupG* or *atpI_rsmG* or expressed from the plasmid pETDuet. Measured CYP450 concentrations were normalized to the measured cell dry weight (cdw). 4pluv is short for: atpI_rsmG::atr2_cyp719a23 nupG::sdh_fiplr.

In our previous work, the oxidation of (−)-matairesinol to (−)-pluviatolide, catalyzed by CYP719A23, was identified as the bottleneck of the reaction cascade, and the concentration of this monooxygenase in the cell was found as the main factor for improvement (Decembrino et al., 2020). In this context, different loci were compared regarding the expression levels of CYP719A23 after chromosomal integration alongside the cytochrome P450 reductase ATR2. It has already been demonstrated that the loci *atpI_rsmG* and *nupG* are suitable for high protein expression in *E. coli*, including a bacterial CYP450 enzyme (Bryant et al., 2014; Englaender et al., 2017; Luelf et al., 2023). The CYP719A23 concentration was only 1.6-fold higher when integrated into the *atpI_rsmG* locus compared to the *nupG* locus (187 ± 21 nmol/g_cdw_ versus 120 ± 5 nmol/g_cdw_), as determined based on the CO-difference spectra after 48 h of expression (Figure 2; Supplementary Figure S3). As a result, the strain with *cyp719a23* and *atr2* genes integrated into the *atpI_rsmG* locus was used for further cloning, and the genes of the faster performing enzymes *Fi*PLR and *Pp*SDH were then integrated into the *nupG* locus, generating the strain 4pluv. Co-expression of the four enzymes in 4pluv resulted in slightly decreased CYP719A23 concentrations (161 ± 27 nmol/g_cdw_). The chromosomal system was then compared to the episomal system using either the plasmid pETDuet_atr2_cyp719a23 alone or together with pACYCDuet_ppsdh_fiplr for co-expression. The CYP719A23 concentration of 208 ± 27 nmol/g_cdw_ after expression from a plasmid with ∼40 gene copies per cell - according to the pET system manual, Novagen - was only to a small degree higher compared to the chromosomal expression with one gene copy integrated into the *atpI_rsmG* locus. Again, co-expression of all four genes led to a marginally decreased CYP450 concentration. The higher metabolic burden imposed on the cell by plasmid-based expression compared to chromosomal expression was reflected in the growth curves of both strains (Supplementary Figure S4). Furthermore, the genetic stability of the two expression systems was compared by passaging both strains every 24 h to fresh agar plates with LB medium but without adding antibiotics. Every 5 days, it was verified that the plasmid-containing strain is still capable of growing on selection medium supplemented with ampicillin and chloramphenicol. After ten passages, the plasmid-containing strain was still able to grow in the medium containing both antibiotics. After 15 passages, no growth was observed anymore. At this time point, for the strain with the chromosomally integrated recombinant genes, the presence of the recombinant genes was verified by amplification via colony PCR.

### 3.2 (−)-Pluviatolide production in resting cells

**FIGURE 3:**
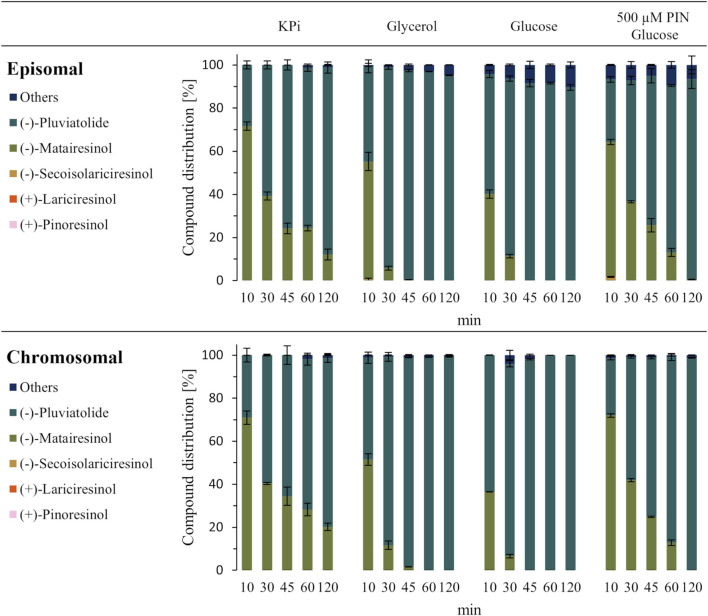
Biotransformation of (+)-pinoresinol (PIN, 200 µM) to (−)-pluviatolide in resting cells (70 g/L cww). Depicted is the compound distribution of the substrate, intermediates and product at different time points for the episomal (top) and chromosomal (bottom) expression systems. The resting cells were resuspended in different solutions, from left: potassium phosphate buffer (KPi, 50 mM, pH 7.5), KPi with 400 mM glycerol, KPi with 200 mM glucose. Finally, the substrate amount was increased to 500 µM (+)-pinoresinol using resting cells in KPi with glucose (right).

Next, the biocatalytic performance of resting cells (70 g/L cww) using either the chromosomal or the episomal system was compared in terms of the conversion of 200 µM (+)-pinoresinol. In both cases, the substrate, as well as the intermediates (+)-lariciresinol and (−)-secoisolariciresinol, were completely depleted after 10 min, when the corresponding signals disappeared in the total ion chromatograms. The accumulation of (−)-matairesinol underlines the CYP-catalyzed reaction as rate-limiting: While the concentration of the final product (−)-pluviatolide increased steadily, 12% and 20% of (−)-matairesinol remained in the solution after 2 h for the episomal and chromosomal systems, respectively (Figure 3).

Both reductive steps from (+)-pinoresinol to (−)-secoisolariciresinol catalyzed by *Fi*PLR require NADPH as a cofactor, which is also needed by ATR2 to enable CYP719A23 activity. Since an increase of the reducing nicotinamide cofactors by the addition of a carbon and energy source could improve the CYP450 performance and thus lead to higher product titers, glycerol and glucose were added at concentrations providing equal carbon equivalents. As expected, the addition of carbon sources led to an accelerated conversion of (−)-matairesinol, which was depleted after 45 min in glucose-containing buffer and after 60 min in glycerol-containing buffer, for both the episomal and chromosomal systems (Figure 3; Supplementary Figure S5). When 500 µM (+)-pinoresinol and glucose as a carbon source were used, again, substrate depletion was completed after 10 min. The formation of (−)-pluviatolide was slowed down, with roughly 25% (−)-matairesinol remaining after 45 min, which was fully converted after 120 min (Figure 3). Importantly, regardless of the conditions tested, the chromosomal system performed at least equally well as the episomal one, with 99% product ratio of (−)-pluviatolide (determined based on the calibration curve, Supplementary Figure S2) achieved if a carbon source was supplemented. Finally, a reduced cell wet weight of 20 g/L with the addition of glucose was compared to the resting cell setup with 70 g/L cww. Here, for both the episomal and chromosomal systems, the biotransformation of 200 µM (+)-pinoresinol to the following intermediates (+)-lariciresinol and (−)-secoisolariciresinol occurred at a similar speed to that observed with higher cell density (Figure 3; Supplementary Figure S6). However complete conversion of (−)-matairesinol to (−)-pluviatolide was only reached after 4 h. This observation provides further evidence that higher concentrations of CYP719A23 (and ATR2) in the sample would lead to faster (−)-matairesinol oxidation. This can be achieved by using either a higher cell concentration or higher levels of CYP and ATR2 expression per cell (see discussion below). Furthermore, recombinant CYP450 enzymes have limited stability. To investigate if the CYP719A23 amount decreases during the biotransformation in resting cells (20 g/L cww), the CYP450 concentration was measured before the reaction, and after the conversion to (−)-pluviatolide (4 h). For the episomal and chromosomal systems, a 21%–37% lower concentration of CYP719A23 was measured after the biotransformation.

### 3.3 (−)-Pluviatolide production in growing cells

Next, the conversion of (+)-pinoresinol to (−)-pluviatolide in growing cells was investigated. Initially, the effect of adding the substrate at different start points after induction was compared for the chromosomal system using the strain 4pluv. It can be reasoned that the time point for the start of biotransformation can influence the metabolic state of growing cells, e.g., by the consumption of cofactors or the presence of toxic intermediates, and thus influence the final product titer. In the stationary phase, cell growth stops, while metabolic activity is maintained, resembling the conditions in resting cells. When (+)-pinoresinol was added 24 h after induction, in the stationary phase, it was depleted 2 h later. However, 41% of (−)-matairesinol remained unconverted 24 h later (48 h after induction) (Supplementary Figure S7). When (+)-pinoresinol was added 2 h after induction and the biotransformation began during the exponential growth phase, the conversion of (−)-matairesinol was improved with (−)-pluviatolide accounting for 78% after 24 h. Having identified 2 h after induction as a suitable time point for substrate addition, this condition was chosen for comparison of the episomal and chromosomal systems and further optimization.

**FIGURE 4:**
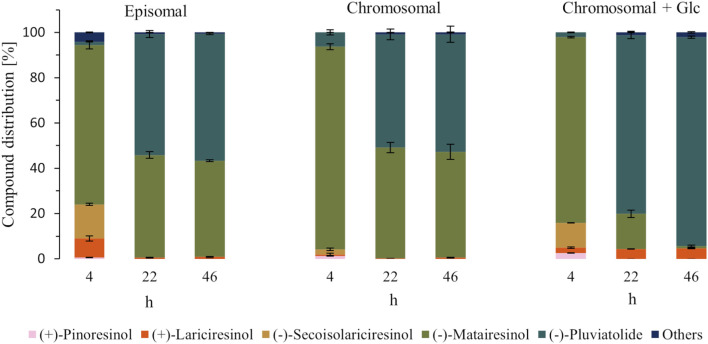
Biotransformation of (+)-pinoresinol (200 µM) to (−)-pluviatolide in growing cells. Depicted is the distribution of the substrate, intermediates and product at different time points during the biotransformation (hours after substrate addition, which occurred 2 h after induction) for the episomal and chromosomal systems, for the latter also with glucose (Glc, 5 g/L) supplemented to the medium.

Also, in the growing cells approach, the two systems performed very similarly, with complete (+)-pinoresinol depletion after 4 h of biotransformation and 53% and 50% (−)-pluviatolide formed after 22 h in the episomal and chromosomal systems, respectively. However, the ratio of (−)-matairesinol and (−)-pluviatolide stagnated between 22 h and 46 h of biotransformation. Since glycerol present in the TB medium was not sufficient for complete (−)-matairesinol conversion, the addition of glucose to the TB medium was tested, as used for the production of raspberry ketone in *E. coli* (Wang et al., 2019). Whereas after 4 h, traces of (+)-pinoresinol and 13% (−)-secoisolariciresinol left unconverted and only 2% (−)-pluviatolide formed, after 22 h, the (−)-pluviatolide ratio increased to 79%. After 46 h, (−)-matairesinol disappeared and (−)-pluviatolide achieved 92% which is the highest product ratio of (−)-pluviatolide obtained in growing cells (Figure 4). The residual 8% were distributed between lariciresinol and other unidentified metabolites not present in the control strain without recombinant genes.

## 4 Discussion

Microbial cell factories are increasingly employed for one- and multi-step biotransformations due to advantages such as cofactor regeneration and improved catalyst stability (Siedler et al., 2011; Yan et al., 2024). In order to increase their efficiency, the distribution of cellular resources between cell growth and product formation, as well as between the genes utilized in a multi-enzyme cascade should be optimized (Saleski et al., 2021). This can be achieved through process and/or enzyme engineering, as well as by adjusting promoter strengths and gene copy numbers (Xu et al., 2017). In this study, we transferred genes encoding the enzymes from the pathway from (+) pinoresinol to (−)-pluviatolide into the chromosome of *E. coli*. We then compared the catalytic performance of both resting and growing cells using different carbon sources, in both chromosomal and episomal systems.

For the episomal system with 70 g/L cww resting cells, the conversions of the substrate and subsequent intermediates are in agreement with our previous study (Decembrino et al., 2020). Specifically, this means i) rapid and complete consumption of (+)-pinoresinol, ii) the accumulation of (−)-matairesinol and iii) the final (−)-pluviatolide titer. Moreover, equal activity was observed for (−)-pluviatolide production in both chromosomal and episomal systems, because the determined concentrations of the rate-limiting enzyme CYP719A23 were quite similar in both systems, and the upstream enzymes *Fi*PLR and *Pp*SDH converted their respective substrates within the first 10 min. At a lower cell concentration of 20 g/L cww resting cells, the difference between the chromosomal and episomal systems was slightly more pronounced (Supplementary Figure S6), indicating higher concentrations of the enzymes in the episomal system with a high number of gene copies compared to the chromosomal system with only one copy of each enzyme. Indeed, though the metabolic flux of the respective cells for the two studied systems might differ, for example, due to the increased metabolic burden imposed by the plasmids, the metabolic flux within the cascade does not differ significantly. This could be explained by the limiting role of the CYP719A23-catalyzed reaction. Complete conversion of the CYP substrate (−)-matairesinol to (−)-pluviatolide was enabled by adding a carbon source, with glucose and glycerol having a very similar effect. Generally, the choice of carbon source can alter the ratio of oxidized to reduced cofactors in the cell (San et al., 2002). Glucose and glycerol differ in their degree of reduction (i.e., the number of electrons available per carbon atom) with four and 4.67 equivalents, respectively (Shams Yazdani and Gonzalez, 2008). The higher reductive power of glycerol has been reported to lead to higher theoretical yields of reduced metabolites to maintain redox balance, making it an interesting choice for biosynthetic pathways consuming NAD(P)H (Nikel et al., 2010; Klein et al., 2016). However, there are also examples for *E. coli* resting cells, where glucose led to higher product yields than glycerol (Tieves et al., 2016). Generally, it has been demonstrated that the addition of glucose to nutrient-deficient *E. coli* cells results in a fast increase in intracellular NAD(P)H levels (Goldbeck et al., 2018; Zhang et al., 2018). Consequently, adding glucose to resting cells has also been reported to improve the conversion of other NADPH-dependent reactions, such as vanillate reduction by a carboxylic acid reductase, reduction of dihydroisoquinolines by an imine reductase and production of ε-caprolactone by a Baeyer-Villiger monooxygenase (Leipold et al., 2013; Nain et al., 2024; Wang et al., 2025).

No difference in substrate conversion has been reported between resting and growing cells in a CYP-catalyzed whole-cell production of 15*β*-hydroxycyproterone acetate (Kiss et al., 2015). However, CYP450 enzymes are considered not very stable and activity may decrease over time, diminishing the achievable product titer in resting cells (Lundemo et al., 2016; Bertelmann and Bühler, 2024). Indeed, in the experiments with 20 g/L of resting cells, a reduction of the CYP719A23 concentration from the start of the biotransformation until the completion of (−)-pluviatolide production after 4 h was observed for both the episomal and the chromosomal systems. This issue might be mitigated by continuous expression in growing cells (Zehentgruber et al., 2010; Bertelmann and Bühler, 2024). In our study, in growing cells, the (−)-pluviatolide production was equally effective in the episomal and chromosomal systems. Similar to resting cells, supplementing the medium with glucose accelerated product formation in growing cells, allowing complete conversion of (−)-matairesinol to (−)-pluviatolide after 45 min and after 48 h, respectively. However, resting cells were faster than growing cells in each step of the cascade. Here, several factors must be considered for this comparison. Biotransformation in growing cells started at low cell densities, with a final cell mass after 48 h of only 6 g/L cdw, in contrast to the 18 g/L cdw (70 g/L cww) used in resting cells throughout the entire process. In the chromosomal system, the substrate conversions rates normalized to the cell mass were 0.072 and 0.058 μmol g^-1^ min^-1^ in resting and growing cells, respectively. Therefore, the productivity per cell mass is comparable in growing cells. Together with the depletion of NAD(P)H by the ongoing anabolism of growing cells and the lower cell and enzyme levels at early stages of cultivation, this can account for the slower substrate and intermediate conversions.

Regardless of the setup tested in this study, i.e., episomal or chromosomal systems in resting or growing cells, the step catalyzed by CYP719A23 was confirmed as the bottleneck of the entire cascade. In general, plant CYP450s are particularly challenging for heterologous expression and implementation in multi-enzyme cascades in *E. coli* due to their low expression levels, low stability and low turnover rates and imperfect membrane-associated organization with their redox partners (Zhou et al., 2021; Li et al., 2024). Common attempts to improve CYP450 expression and activity in prokaryotic hosts, such as N-terminal truncations and modifications, the screening of different redox partners or redox partner stoichiometries and fusing with ATR2 have already been applied to CYP719A23 in our previous study (Decembrino et al., 2020). In the present study, the two loci *nupG* and *atpI_rsmG* were compared to achieve the most appropriate expression of CYP719A23 and the redox partner ATR2. The CYP719A23 concentration was higher in the *atpI_rsmG* locus, which is well explained by the gene dosage effect, as *atpI_rsmG* is located closer to the origin of replication (ori) than the *nupG* locus. Other studies have also shown that expression of a fluorescence reporter protein was higher from the *atpI_rsmG* locus than from the *nupG* integration site, whereas only minor differences were observed for the expression of the bacterial CYP154E1 (Goormans et al., 2020; Luelf et al., 2023). Gene expression also depends on other factors that cannot be excluded, such as the extent of supercoiling and DNA gyrase distribution, the local chromosomal context, transcriptional interference or read-through (Bryant et al., 2014; Scholz et al., 2019; Goormans et al., 2020).

Here, the expression of CYP719A23 from the *atpI_rsmG* locus was comparable to the expression from the pETDuet vector. Plasmid-based expression goes along with a significantly higher copy number of a gene compared to one copy on the chromosome. However, this can also result in misfolded proteins and inclusion body formation in *E. coli* (Iafolla et al., 2008). In particular, for CYP450 enzymes, insufficient heme incorporation can result in the accumulation of nonfunctional enzyme (Ge et al., 2023; Hu et al., 2023). Chromosomal expression might therefore even lead to elevated functional protein content in the cell (Englaender et al., 2017; Luelf et al., 2023). The ratio of reductase to CYP may also influence CYP activity (Bassard et al., 2017), and additional copies of both CYP and reductase will be tested in future studies to increase CYP activity. In some cases, a higher expression level of CYP450 was beneficial for achieving higher product titers (Vazquez-Albacete et al., 2017; Poborsky et al., 2023). However, a balanced ratio of the CYP450 enzyme to its redox partner protein, as well as to other enzymes participating in the cascade, may be even more important than the CYP450 concentration, as described for the biosynthesis of oxygenated taxanes in an *E. coli* strain (Biggs et al., 2016). This might become particularly important for the desired extension of the pathway to (−)-podophyllotoxin.

### 4.1 Conclusion

Herein, we improved the production of (−)-pluviatolide from (+)-pinoresinol in recombinant *E. coli* by combining gene integration into the chromosome and process engineering. The productivity of resting cells for (−)-pluviatolide synthesis was similar in the chromosomal and episomal systems. The addition of glucose to the buffer or medium led in both resting and growing cells to complete conversion of the critical intermediate (−)-matairesinol to (−)-pluviatolide with final product ratios of 99% and 92%, respectively. Further steps to overcome the bottleneck of the cascade might include metabolic engineering approaches for improved cofactor supply or the addition of further copies of CYP719A23 and/or ATR2 into the chromosome. For the latter, it remains to be investigated if the trade-off between the increased metabolic burden and elevated enzyme concentrations will have a beneficial effect compared to the benchmark set in this study. This work is an important next step towards the heterologous biosynthesis of (−)-podophyllotoxin. In this context, the application of the chromosomal system, as well as the option to switch between resting and growing cells, could become valuable during the extension of the cascade towards (−)-podophyllotoxin, with two more CYP450 enzymes and two methyltransferases present in the pathway competing for cofactors.

## Data Availability

The raw data supporting the conclusions of this article will be made available by the authors, without undue reservation.
