# Supplementary material for: Integrated biosynthesis of the lignan (-)-pluviatolide in resting and growing E. coli cells
Source: Front Bioeng Biotechnol. 2026 Jan 16;14:1716646. doi: 10.3389/fbioe.2026.1716646 (PMC12855525; doi:10.3389/fbioe.2026.1716646)
Supplement: Supplementary file 1 [file DataSheet1.pdf]

Supplementary Material

1 Supplementary Tables

Table S1 Genes used in this study for biotransformation. All genes are codon-optimized for *E. coli* and *cyp719a23* and *atr2* are truncated as described before. Please refer to the listed sources for the exact sequences of the genes.

Gene	Name	Origin	Accession number (Genbank)	Source
fiplr	Pinoresinol-lariciresinol reductase	*Forsythia x intermedia*	U81158.1	[1]
ppsdh	Secoisolariciresinol dehydrogenase	*Podophyllum pleianthum/Dysosma pleiantha*	KC551922.1	[2]
cyp719a23	Pluviatolide synthase	*Podophyllum hexandrum*	KC110997.1	[2]
atr2	NADPH-cytochrome P450 reductase 2	*Arabidopsis thaliana* Columbia	NM_119167.4	[3]

Table S2 *E. coli* strains used in this study.

Strain	Purpose	Source
DH5α	Cloning purposes	Clontech
C41(DE3) Overexpress	Genome engineering, control strain for expression and biotransformation	Lucigen
C41(DE3) pETDuet pACYCDuet	Control strain for expression and biotransformation	This study
C41(DE3) pETDuet_atr2_cyp719a23	Expression	This study
C41(DE3) pETDuet_atr2_cyp719a23 pACYCDuet_ppsdh_fiplr	Expression and biotransformation	This study
C41(DE3) nupG::atr2_cyp719a23	Expression	This study
C41(DE3) atpI_rsmG::atr2_cyp719a23	Expression and genome engineering	This study
C41(DE3) nupG::ppsdh_fiplr atpI_rsmG::atr2_cyp719a23 (4pluv)	Expression and biotransformation	This study

Table S3 Plasmids used in this study.

Plasmid	Purpose	Source
pCDFDuet_ppsdh_fiplr	Cloning	[2]
pACYCDuet	Cloning and expression	Novagen
pACYCDuet_ppsdh_fiplr	Cloning and expression	This study
pETDuet	Cloning and expression	Novagen
pgRNA_atpI_rsmG	Cloning	[4]
pgRNA_nupG	Cloning	[4]
pgRNADuet_atpI_rsmG	Cloning	This study

pgRNADuet_nupG	Cloning	This study
pETDuet_atr2_cyp719a23	Cloning and expression	[2]
pgRNADuet_nupG_ppsdh_fiplr	Cloning	This study
pgRNADuet_nupG_atr2_cyp719a23	Cloning	This study
pgRNADuet_atpI_rsmG_atr2_cyp719a23	Cloning	This study
pEcCas	Cloning	[5]

Table S4 Primers used in this study.

**Name**	**Sequence (5'→ 3')**	**Source**
Linearization of pgRNA and introduction of overhangs to the respective homology arms		
pgRNA atpI_rsmG rev	GTGTTACTGGTGGTGGCTTCAAAAAAAGCACCGACTC	[6]
pgRNA atpI_rsmG fw	CTCATGTTGCACCTGACGGGGATAACGCAGGAAAGAAC	[6]
pgRNA nupG rev	GATTAACCCAAGCGTTGGCATTCAAAAAAAGCACCGACTC	This study
pgRNA nupG fw	CACCGCGAATATAAGCGCTAGGGATAACGCAGGAAAGAAC	This study
Amplification of homology arms, primers marked with an asterisk were also used for Fusion PCR		
HA1 nupG fw *	TGCCAACGCTTGGGTTAATC	[4]
HA1 nupG rev	GGATGGTCAGAATGAACAGGG	[4]
HA2 nupG fw	CTAACGGCTTCGGCTGTATC	[4]
HA2 nupG rev *	TAGCGCTTATATTCGCGGTG	[4]
HA1 atpI_rsmG fw *	GCCACCACCAGTAACAC	[4]
HA1 atpI_rsmG rev	TGATCGAACAGGGTTAGC	[4]
HA2 atpI_rsmG fw	CAGCCAATGATGGTTCTTAGC	[4]
HA2 atpI_rsmG rev *	CGTCAGGTGCAACATGAG	[4]
Amplification of pETDuet multiple cloning sites and introduction of overhangs to the respective homology arms		
pETDuet fw_HA2 nupG	GATACAGCCGAAGCCGTTAGTTAATGCGCCGCTACAGG	[4]
pETDuet rev_HA1 nupG	CCCTGTTCATTCTGACCATCCTATAGGCGCCAGCAACC	[4]
pETDuet fw_HA2 atpI_rsmG	GCTAACCCTGTTCGATCATTAATGCGCCGCTACAGG	[4]
pETDuet rev_HA1 atpI_rsmG	GCTAAGAACCATCATTGGCTGTATAGGCGCCAGCAACC	[4]
Verification of integration events		
atpI_rsmG fw	TCAGCGGCAAGAATACC	[6]
atpI_rsmG rev	TCCTGAAGCCCATTTCAC	[6]
nupG fw	GAAGCGATGTTCCTTGTG	This study
nupG rev	CAGAGTTTCCCGTCAGTG	This study

Table S5 LC-MS gradient used in this study.

Time [min]	Solvent B [%]
0.01	20
2	40
5	45
25	48
32.5	100
35	100
35.01	20
40	20

2 Supplementary Figures

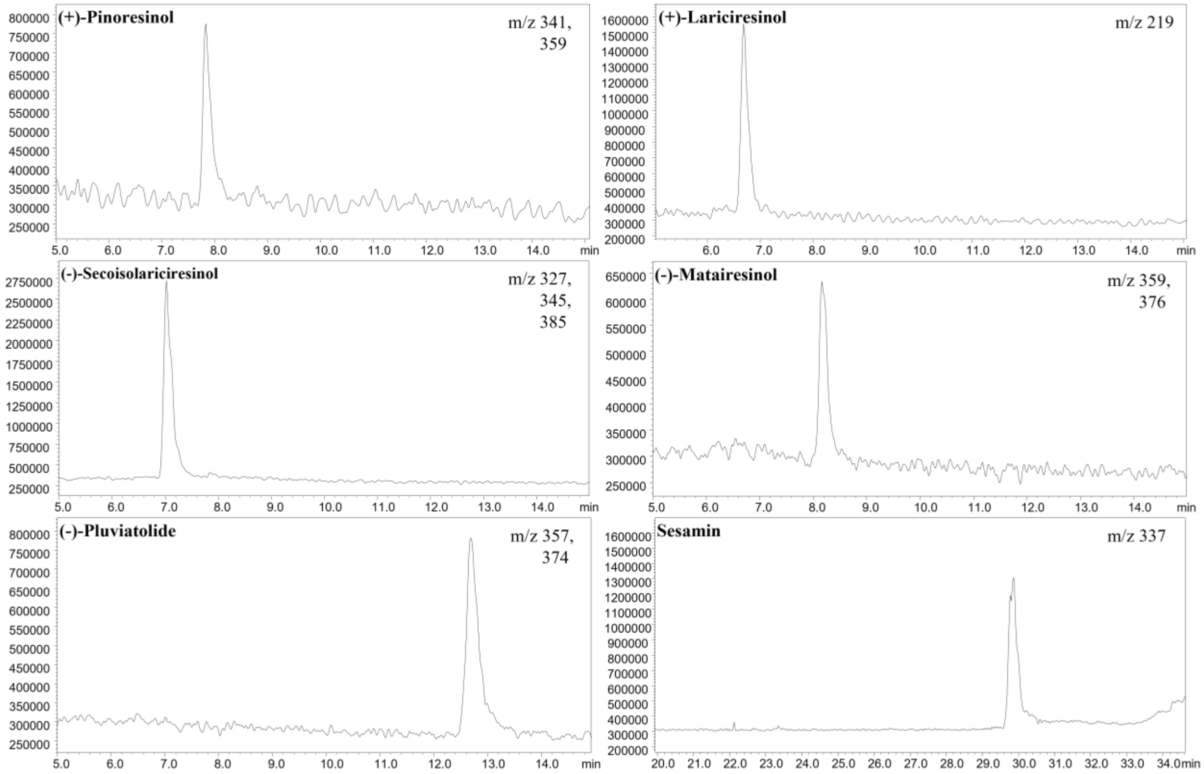

Figure S1 Total ion chromatograms (TIC) of authentic references of all analytes in the biocatalytic cascade from (+)-pinoresinol to (-)-pluviatolide and the internal standard. The most abundant m/z values observed are given in the top right corner of the respective TIC for each compound. The retention times are as follows in order of elution: (+)-lariciresinol 6.7 min, (-)-secoisolariciresinol 7.0 min, (+)-pinoresinol 7.8 min, (-)-matairesinol 8.2 min, (-)-pluviatolide 12.7 min, sesamin (internal standard) 29.9 min.

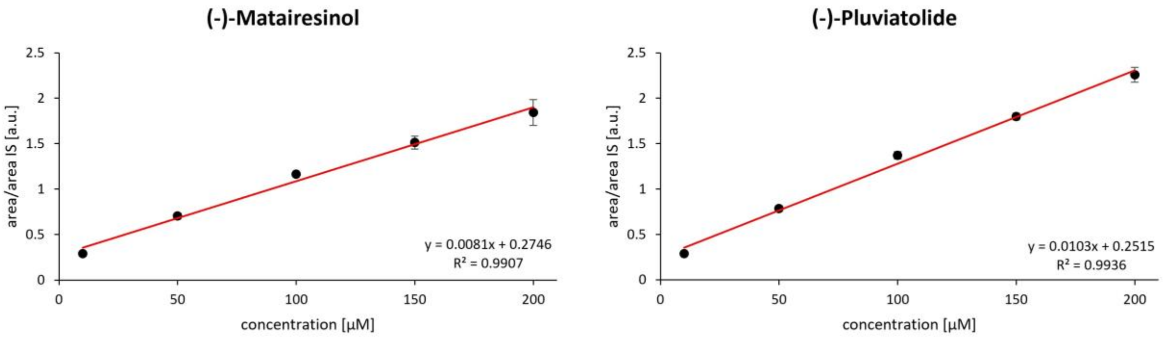

Figure S2 Internal standard (IS) calibration curves for the quantification of (-)-matairesinol and (-)-pluviatolide.

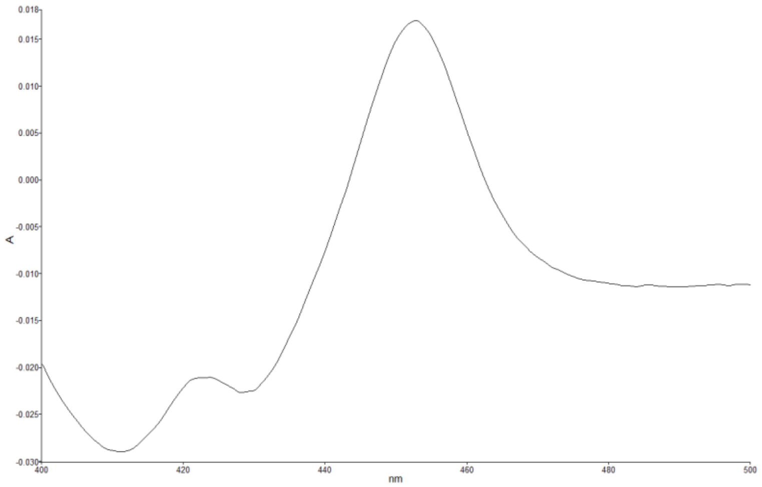

Figure S3 Representative CO-difference spectrum. Depicted is the spectrum of CYP719A23 after expression of the gene integrated into the *atpI_rsmG* locus of *E. coli* C41(DE3) with the characteristic maximum at 450 nm.

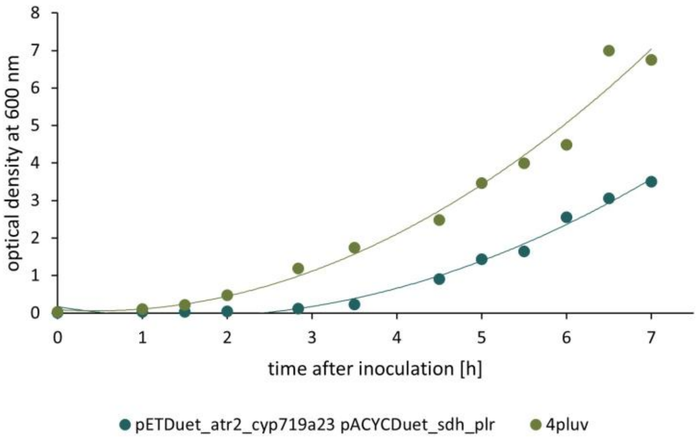

Figure S4 Growth curves of the recombinant *E. coli* C41(DE3) strains with episomal (dark green) or chromosomal (light green) gene expression after inoculation of the main cultures.

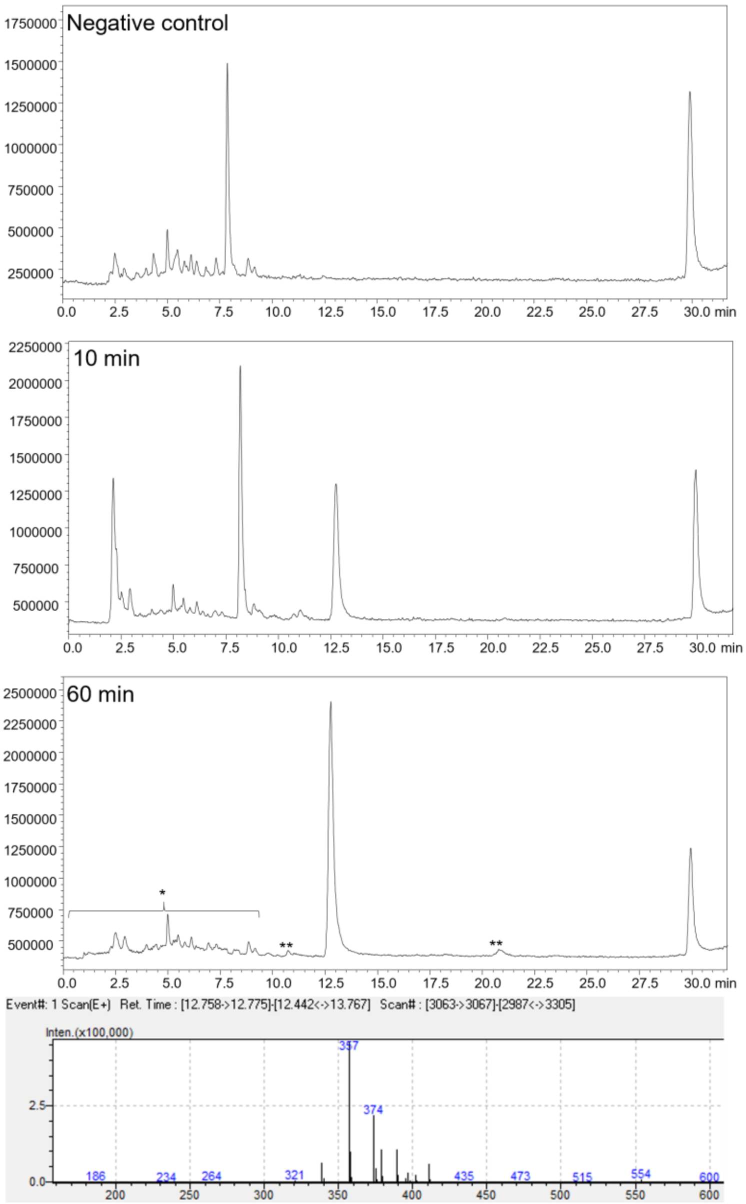

Figure S5 Representative chromatograms for the production of (-)-pluviatolide from (+)-pinoresinol using 70 g/L cww resting cells of *E. coli* with added carbon source. Top: TIC of the *E. coli* C41(DE3) wild-type control strain without recombinant genes after 60 min. (+)-Pinoresinol (retention time 7.8 min) was not converted. Middle: TICs of the biotransformation of (+)-pinoresinol. After 10 min, the substrate was depleted and (-)-matairesinol (8.2 min) and (-)-pluviatolide (12.7 min) were formed. After 60 min, (-)-matairesinol was completely converted to (-)-pluviatolide. The peak at 29.9 min belongs to sesamin used as internal standard. Signals marked with asterisks result from the cellular background (*), also present in the negative control, or unidentified metabolites (**). Bottom: Extracted ion chromatogram at 12.7 min of the TIC above (60 min) showing the produced (-)-pluviatolide.

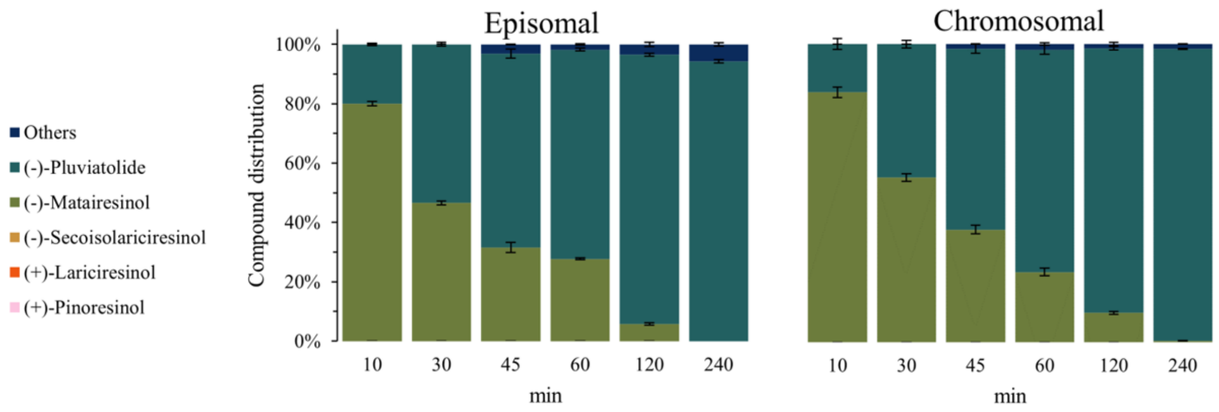

Figure S6 Biotransformation of (+) pinoresinol to (-)-pluviatolide in resting cells (20 g/L cww). The cells were resuspended in potassium phosphate buffer (KPi) with 200 mM glucose. Depicted is the compound distribution of the substrate, intermediates and product at different time points for the episomal (left) and chromosomal (right) expression systems.

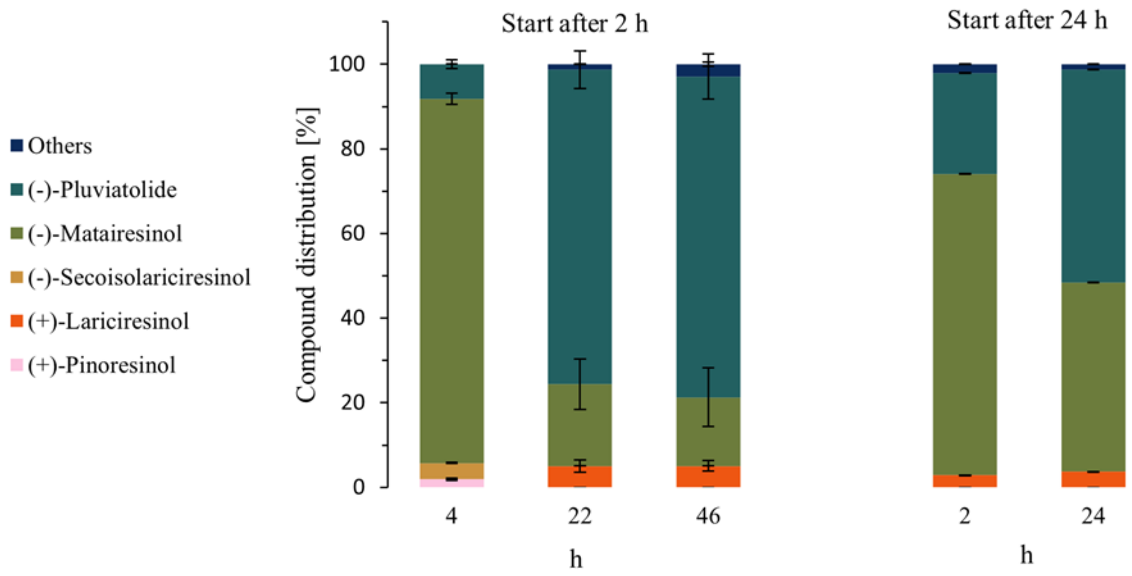

Figure S7 Start of biotransformation in growing cells at different time points. Left: (+)-pinoresinol was added 2 h after induction. The distribution of substrate, intermediates and product is shown at 4 h, 22 h and 46 h after the start of the biotransformation, respectively. Right: (+)-pinoresinol was added 24 h after induction and samples were drawn 2 h and 24 h after the substrate feed.

References

[1] Ricklefs, E., Girhard, M., and Urlacher, V.B. (2016). Three-steps in one-pot: whole-cell biocatalytic synthesis of enantiopure (+)- and (-)-pinoresinol via kinetic resolution. *Microb. Cell Fact.* 15**,** 78. doi: 10.1186/s12934-016-0472-0

[2] Decembrino, D., Ricklefs, E., Wohlgemuth, S., Girhard, M., Schullehner, K., Jach, G., et al. (2020). Assembly of plant enzymes in *E. coli* for the production of the valuable (-)-podophyllotoxin precursor (-)-pluviatolide. *ACS Synth. Biol.* 9(11)**,** 3091-3103. doi: 10.1021/acssynbio.0c00354

[3] Kranz-Finger, S., Mahmoud, O., Ricklefs, E., Ditz, N., Bakkes, P.J., and Urlacher, V.B. (2018). Insights into the functional properties of the marneral oxidase CYP71A16 from *Arabidopsis thaliana*. *Biochim. Biophys. Acta Proteins Proteom.* 1866(1)**,** 2-10. doi: 10.1016/j.bbapap.2017.07.008

[4] Luelf, U.J., Böhmer, L.M., Li, S., and Urlacher, V.B. (2023). Effect of chromosomal integration on catalytic performance of a multi-component P450 system in *Escherichia coli*. *Biotechnol. Bioeng.* 120(7)**,** 1762-1772. doi: 10.1002/bit.28404

[5] Li, Q., Sun, B., Chen, J., Zhang, Y., Jiang, Y., and Yang, S. (2021). A modified pCas/pTargetF system for CRISPR-Cas9-assisted genome editing in *Escherichia coli*. *Acta Biochim. Biophys. Sin.* 53(5)**,** 620-627. doi: 10.1093/abbs/gmab036

[6] Luelf, U.J., Wassing, A., Böhmer, L.M., and Urlacher, V.B. (2024). Plasmid-free production of the plant lignan pinoresinol in growing *Escherichia coli* cells. *Microb. Cell Fact.* 23(1)**,** 289. doi: 10.1186/s12934-024-02562-3
